# Efficacy of a Self-timed Trial of Laparoscopic Surgical Training Using a Dry Box

**Published:** 2018-02-08

**Authors:** Kenro Chikazawa, Hiroyuki Kanao, Tomonori Hada, Sachiho Netsu, Tomoyuki Kuwata, Ryo Konno

**Affiliations:** ^a^Jichi Medical University, Saitama Medical Center, Saitama, Japan; ^b^Department of Gynecology, Cancer Institute Hospital, Tokyo, Japan; ^c^Department of Obstetrics and Gynecology, Kurashiki Medical Center, Okayama, Japan

**Keywords:** self-timed trial, surgical training, dry box training, experiment, suturing

## Abstract

**Objective:** This study evaluated the self-timed trial training for laparoscopic suturing. **Methods:** The set task involved grasping the suture, aligning the needle with a needle holder, passing the suture, making 3 knots, holding the 2 tails of the suture with one grasper, and cutting them. Trainees were given an instruction for suturing and reducing their suturing time. The same instruction was given 3 months later. Suturing times for the first and second trials and the last trial after the second instruction of the 9 trainees were measured. **Results:** Their mean suturing times were statistically significantly shorter after instruction (before instruction: 276.7 ± 43.4 seconds, after instruction: 177.4 ± 46.1 seconds; *P* = .0035). Four trainees were trained twice during the second instruction. Their suturing times were shorter than those of the other trainees, and the standard deviation decreased (120.5 ± 21.2 seconds, *P* = .017). **Conclusion:** A self-timed trial training for laparoscopic suturing using a dry box makes training interesting and motivates trainees.

There has been an increasing use of minimally invasive operation with a concurrent decrease in the number of open cavity operations.^1^ There has also been a reduction in the number of junior residents participating in operations.^2^ Many studies about commonly performed operations report favorable patient outcomes.^3^^-^5 Yet, surgeons must train residents effectively while keeping associated costs low.

In laparoscopic operation, training is essential for improving one's surgical skill.^6^ Training with a dry box has been reported necessary and effective.^7^ Of course, it is desirable to learn by performing an actual operation. However, in laparoscopic operations, many surgeons have difficulty educating trainees during the actual operation because it is challenging for the mentor, who uses an assistant to guide the scope, to also guide the trainee's hands; therefore, there have been reports of simulation.^8^ If a trainee causes severe bleeding that is not easy to stop using an energy device, patients must undergo open laparotomy. Before starting training during an actual operation, residents should be familiar with using a grasper, forceps, and needle holder in a simulator or dry box for laparoscopic operation.

Another problem is associated with training during an actual operation. If an institution has many trainees, they will obtain little surgical experience. There are many simulators for virtual reality training of laparoscopic operation, but they are very expensive^8^; dry box training costs a few hundred dollars. We assume that training with a dry box is efficient. Indeed, in an earlier study, both box and virtual reality simulator trainers were equally effective in teaching laparoscopic skills.^8^

A previous study reported that suturing training improved residents’ surgical skill.^9^ On the basis of our experience, we think that training shortens the suturing time, improves the trainee's skill for moving the grasper, reduces the touching error, and refines the tracks of the needle holder and grasper. Suturing improves hand-eye coordination, which is important during laparoscopic operation.^10^ In addition, training improves hand-eye coordinated movement, so surgeons can use their hands more skillfully during open laparotomy.

We hypothesized that self-timed trial training for laparoscopic suturing with a dry box would be useful for improving skills required for laparoscopic operations. This training method does not require a complex program, test, or curriculum. Here, we describe our attempt at using a self-timed trial and the results of our residents’ training.

## METHODS

A box trainer (Lapatore K, Kotobuki Medical Training Tools, Saitama, Japan) was used in this study. A television image showing the inside of the box was visualized through a video camera (JVC, Tokyo, Japan). We also used the On-Lap 1303H/J monitor (Tekwind Co., Ltd., Tokyo, Japan). Laparoscopic instruments, including a needle holder (ENDOPATH, Ethicon, Inc., Tokyo, Japan), Maryland forceps and scissors (Ethicon, Inc.), and surgical thread (1-0 vicryl CT-1 25 cm, Ethicon Inc.), were used.

Trainees were residents of the laparoscopy department of obstetrics and gynecology. Their years of experience as a doctor are shown in Table 1.

The set task was grasping the suture, aligning the needle with a needle holder, passing the suture, making 3 knots, holding the 2 tails of the suture with one grasper, and cutting them (Video 1). The trainer gave a lecture on how to hold the needle and tie the knots. The lecture was divided into 3 sections (holding the needle, tying the knot, and Z suturing and continuous suturing). The knots were tied using the following method. First, a P-loop was created with the long tail of the suture; second, the assisting grasper was placed over the loop, and the suture was wrapped around the instrument in an overwrapping direction; and finally, the free suture end was grasped and pulled through, creating a square first throw. This procedure was repeated with the P-loop under the loop for the second throw, thus completing the square knot. The third throw was made over the loop.^11^

The trainer provided the instruction for 3 hours. Trainees’ times for the first trial were measured. Next, the trainees received a lecture about laparoscopic suturing. Then, they were trained on how to grasp the suture and make a knot for 1 hour each session. A Japanese textbook on laparoscopic and hysteroscopic operations was used as a reference teaching material.^11^ Trainers also created a Microsoft PowerPoint presentation on the basis of this textbook. Details of holding the needle and knot tying were almost accordant to these videos on YouTube: holding needle https://www.youtube.com/watch?v=o6a1oML9EP4 and knot tying: https://www.youtube.com/watch?v=uBsPS_81uJ4. Trainees practiced the task for the timed trial for 1 hour. They were instructed to shorten their suturing time. At the end of the training, trainees’ time for performing the task was measured again. The same instruction was given 3 months later.

Suturing times for the first and second trials, and the last trial after the second instruction, were measured. Trainees’ suturing times 3 months after the first instruction were measured to evaluate the significance and effect of giving the same lecture twice for the same trainees. The maximum time allowed to perform the suturing and knot tying tasks was 3 minutes in all trials.

Continuous data were compared using the Student *t*-test. Additionally, simple regression analysis was used to determine the correlation between changes in the suturing time and years of experience as a doctor. Statistical analysis was performed using the standard Excel setup for Windows (Microsoft Corp., Redmond, Wash). For all statistical tests, a *P* value <.05 was considered significant.

## RESULTS

Suturing times of the 9 trainees were measured (Table 1). Their mean suturing times were statistically significantly shorter after the second instruction. The mean suturing times before and after instruction were 276.7 ± 43.7 and 177.4 ± 46.1 seconds, respectively (*P* = .0035). Four trainees were trained twice during the second instruction. They continued self-timed trials for 3 months. Their times were shorter than the other trainees, and the standard deviation decreased (120.5 ± 21.2 seconds; *P* = .017).

In the simple regression analysis, there was a negative correlation between the first trial time and years of experience as a doctor. However, there was a negative correlation between reducing the trial time after instruction and years of experience as a doctor. Unskilled doctors were able to shorten their trial times.

## DISCUSSION

We found that the self-timed trial and instruction for shortening the suturing time were effective, especially for unskilled doctors. Of course, it is easy to train unskilled trainees in any medical field, including sports medicine. However, there are few studies about laparoscopic dry training. Dry box training can be performed at a low cost, and it does not require a large-scale training center^12^; only a dry box is needed. In our experience, trainees practice suturing without purpose, so the suturing time does not decrease. However, we had trainees undergo training aimed at reducing the suturing time, and trainees’ suturing times were shortened gradually. Presenting trainees with the goal of decreasing the suturing time during the task was important.

Four trainees’ suturing time was shorter than the others. They were able to continue the self-timed trial; thus, they improved their skill. The decreased SD meant that their suturing times had converged at the same skill level. Continuing training can improve trainees’ skill with moving the grasper. We thought that the time trial was useful for keeping trainees motivated, which is important in skill acquisition.^13^ Time-trial training can be performed in other fields, including sports (eg, hill climbing, gymkhana, and dirt trials). Our time trial was applied to laparoscopic suturing training. Decreasing the suturing time gave trainees the feeling of achievement. A time trial makes training interesting, and it motivates trainees. Continuing monotonous training is difficult. Therefore, mentors should make training interesting. If there are other trainees, mentors can have them compete to reduce their suturing time. The decreased time also meant that the trainee's skill of moving the grasper had improved; thus, the touching error was reduced, and tracks of the needle holder and grasper became refined.

This same time-trial training can be applied to another product and training method. In Japan, some doctors improve their laparoscopic skills by using graspers to create origami in a timed trial (https://www.youtube.com/watch?v=iAJixbs8NUY). A past study reported that a virtual reality system was useful, but the environment is artificial and relatively game-like.^14^ Another study reported that a video trainer system like our dry box was better than a virtual reality system for retaining skill.^15^

We educated trainees, using only a dry box, which was inexpensive. Both dry box training and virtual reality simulators are equally effective means of teaching laparoscopic skills.^8^ These training methods are important for performing the initial laparoscopic operation, and they significantly improve performance better than using conventional instruments.^16^ Moreover, this dry box training enables training to be performed with direct vision, with a video camera. A previous study used dry box training effectively and efficiently for junior-level residents.^17^ Manual surgical skill was easier to improve than the skill of hand-eye coordination.^16^ This training method can also be applied to surgeons with different levels of experience.

Moreover, this time trial can be used to evaluate the simple and basic skill of using laparoscopic graspers, forceps, and needle driver. No complicated computer systems or programs are required for evaluation.^18^^-^19 This training method only requires the trainers’ time. Residents can determine their own learning curve by their recording time, and they can improve their skill quickly. Specifically, beginner surgeons experienced more stress than skilled surgeons^20^; thus, a self-timed trial may help residents reduce their preoperative stress if they are able to decrease their trial time.

Our study has limitations to consider. We only evaluated time, not the accuracy of the suturing. The training was aimed at refining trainees’ movement of the forceps, grasper, needle holder, and speed of knot tying. The quality aspects of suturing, such as pitch, bite, and tissue damage, were not evaluated. A future study about the operative time and occurrence of bleeding may be needed. In our study, trainees used a video monitor. However, a recent study reported that trainees could train more effectively under direct vision than indirect visualization.^16^ Therefore, a time trial with direct and indirect visualization may need to be studied.

In conclusion, we suggest self-timed trial training for laparoscopic suturing with a dry box. It makes training interesting and motivates trainees. It is also an inexpensive way to quickly evaluate trainees.

## Figures and Tables

**Table 1:** Examinees’ years of experience as a doctor and trial times

Examinee	Years of experience as a doctor	Before instruction(s)	After instruction(s)	After the second instruction(s)
1	8	190	244	110
2	5	300	112	
3	5	300	190	157
4	8	300	250	105
5	3	300	203	
6	9	200	135	110
7	3	300	170	
8	3	300	163	
9	3	300	130	
Mean		276.7	177.4	120.5
SD		43.7	46.1	21.2
*P*			0.0035	0.017
